# Supplementary material for: Low Prevalence of HLA-G Antibodies in Lung Transplant Patients Detected using MAIPA-Adapted Protocol
Source: Int J Mol Sci. 2023 Nov 18;24(22):16479. doi: 10.3390/ijms242216479 (PMC10671704; doi:10.3390/ijms242216479)
Supplement: Supplementary file 1 [file ijms-24-16479-s001.zip › Figures S1-S5.pdf]

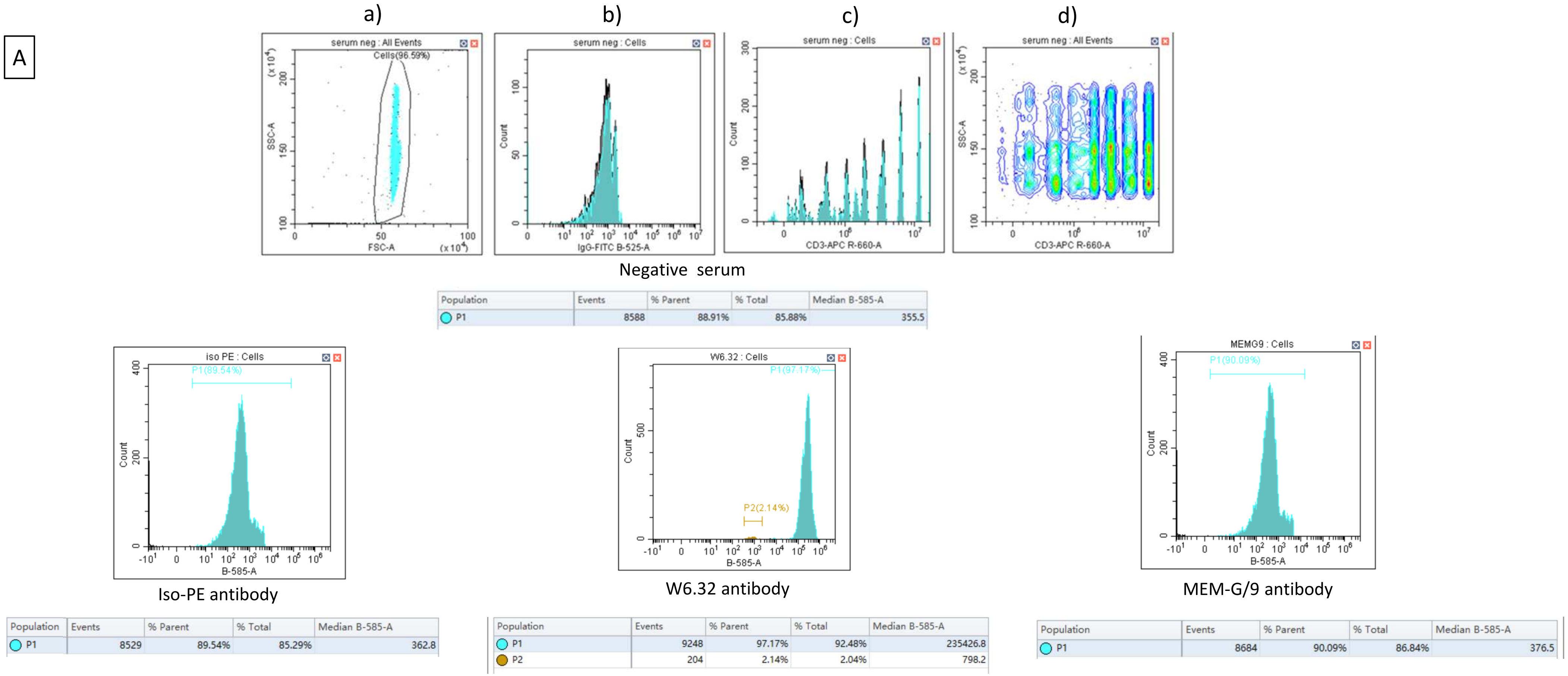

Negative serum

Population	Events	% Parent	% Total	Median B-585-A
P1	8588	88.91%	85.88%	355.5

Iso-PE antibody

Population	Events	% Parent	% Total	Median B-585-A
P1	8529	89.54%	85.29%	362.8

W6.32 antibody

Population	Events	% Parent	% Total	Median B-585-A
P1	9248	97.17%	92.48%	235426.8
P2	204	2.14%	2.04%	798.2

MEM-G/9 antibody

Population	Events	% Parent	% Total	Median B-585-A
P1	8684	90.09%	86.84%	376.5

Supplementary figure 1: cross-reaction of MEM-G9 with other HLA I molecules such as HLA-A and/or HLA-B and/or HLA-C linked to reagent beads from A/ One Lambda Labscren HLA class I Single Antigen and B/ Immucor Lifecode HLA class I Single Antigen using Flow Cytometry.

Luminex Protocol was Adapted for DxFlex. Monoclonal antibodies MEM-G/9 conjugated to PE (ThermoFisher, ref MA1-19643), W6.32 anti-HU HLA-ABC conjugated to PE (eBioscience), and control isotype mouse IgG conjugated to PE (BioLegend) were diluted to the recommended concentration of use (5 micrograms/mL) in negative serum. These samples were processed following the initial steps of the One Lambda (OL) Single Antigen protocol and Immucor Single Antigen protocol. In summary, 5 microL of class I identification beads were incubated with 20 microL of the sample for 30 minutes with agitation. Subsequently, five washes were performed using OL Wash Buffer or Immucor Wash Buffer. The beads were then resuspended in PBS, and the suspension was analyzed using flow cytometry on the DxFlex. Gating strategy for MEM-G/9 cross-reactivity measurements were the following : Identification of beads on the level of forward scatter and side scatter (a). Measurement of the MFI on beads surface (b) Identification of beads on the PCA chanel (c) Identification of beads on the level side scatter and PCA chanel (d).

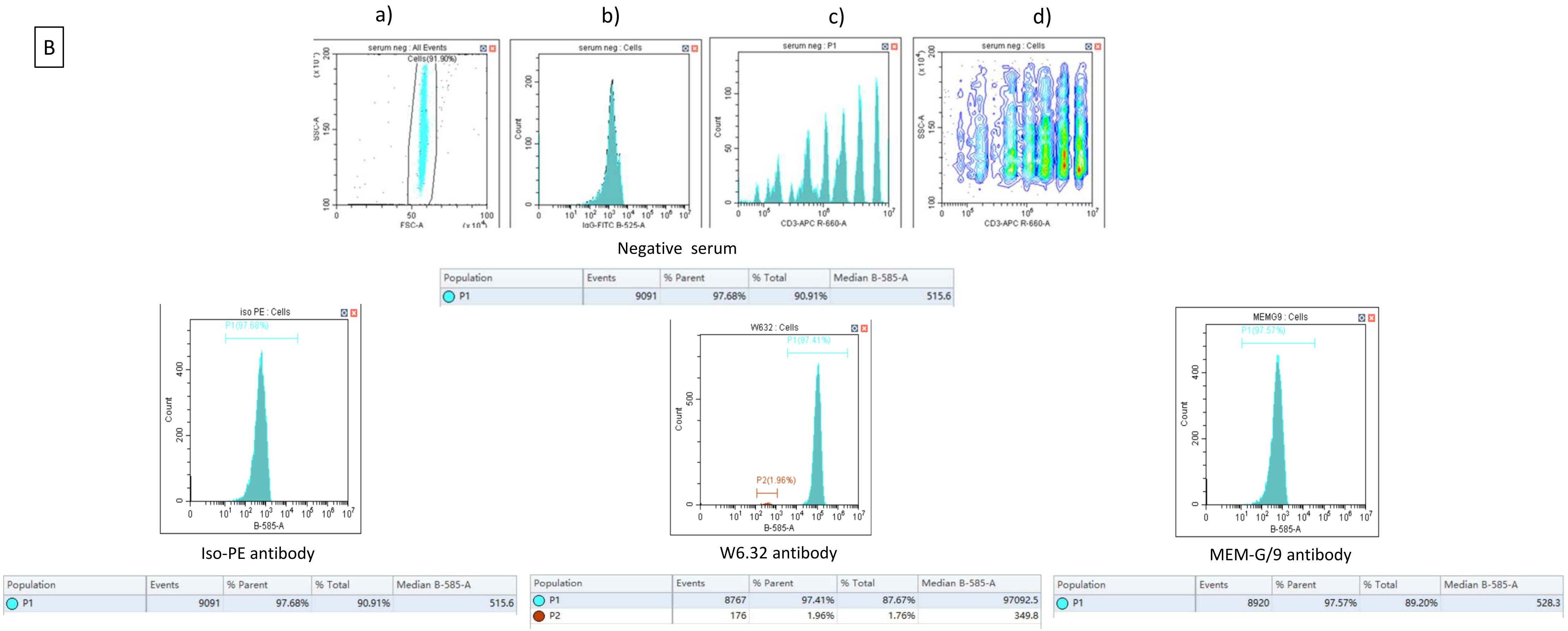

Negative serum

Population	Events	% Parent	% Total	Median B-585-A
P1	9091	97.68%	90.91%	515.6

Iso-PE antibody

Population	Events	% Parent	% Total	Median B-585-A
P1	9091	97.68%	90.91%	515.6

W6.32 antibody

Population	Events	% Parent	% Total	Median B-585-A
P1	8767	97.41%	87.67%	97092.5
P2	176	1.96%	1.76%	349.8

MEM-G/9 antibody

Population	Events	% Parent	% Total	Median B-585-A
P1	8920	97.57%	89.20%	528.3

Supplementary figure 1: cross-reaction of MEM-G9 with other HLA I molecules such as HLA-A and/or HLA-B and/or HLA-C linked to reagent beads from A/ One Lambda Labscren HLA class I Single Antigen and B/ Immucor Lifecode HLA class I Single Antigen using Flow Cytometry.

Luminex Protocol was Adapted for DxFlex. Monoclonal antibodies MEM-G/9 conjugated to PE (ThermoFisher, ref MA1-19643), W6.32 anti-HU HLA-ABC conjugated to PE (eBioscience), and control isotype mouse IgG conjugated to PE (BioLegend) were diluted to the recommended concentration of use (5 micrograms/mL) in negative serum. These samples were processed following the initial steps of the One Lambda (OL) Single Antigen protocol and Immucor Single Antigen protocol. In summary, 5 microL of class I identification beads were incubated with 20 microL of the sample for 30 minutes with agitation. Subsequently, five washes were performed using OL Wash Buffer or Immucor Wash Buffer. The beads were then resuspended in PBS, and the suspension was analyzed using flow cytometry on the DxFlex. Gating strategy for MEM-G/9 cross-reactivity measurements were the following : Identification of beads on the level of forward scatter and side scatter (a). Measurement of the MFI on beads surface (b) Identification of beads on the PCA chanel (c) Identification of beads on the level side scatter and PCA chanel (d).

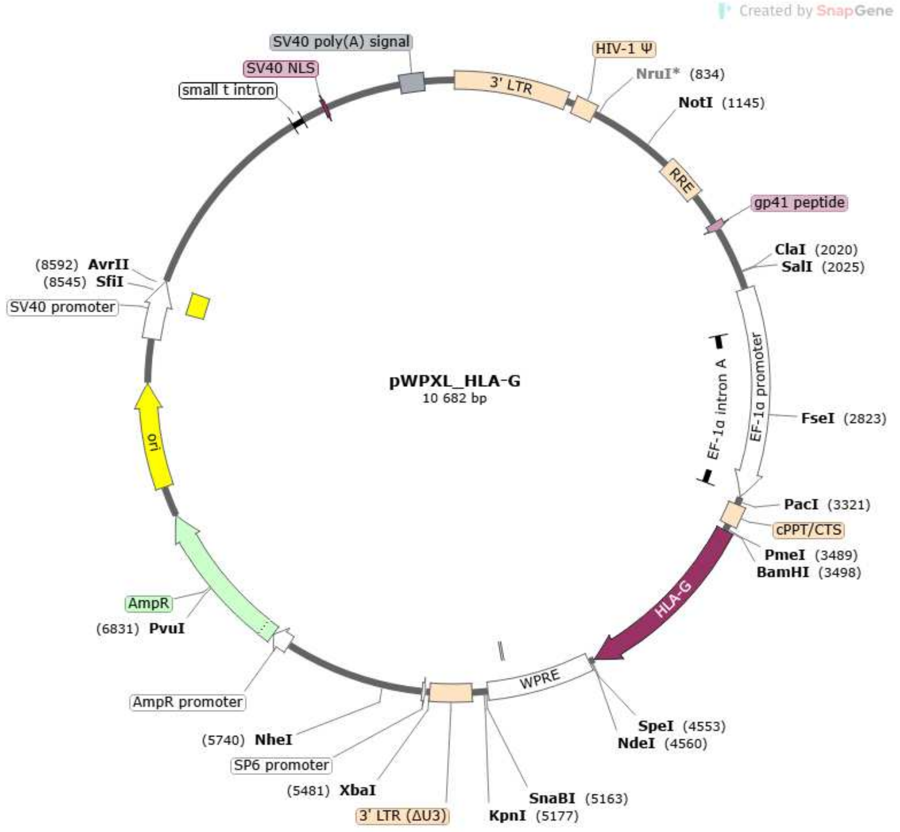

Supplementary figure 2: Plasmid map of the pWPXL_HLA-G construct

>CDS_HLA-G_010101
ATGGTGGTCATGGCGCCCCGAACCCTCTTCCTGCTGCTCTCGGGGGCCCTGACCCTGACCGAGACCTGGGCGGGCTCCCACTCCATGAGGTATTTCAGCGCCGCCGTGTCCCGGCCCGGCCGCGGGGAGCCCCGCTTCATCGCCATGGGCTACGTGGA
CGACACGCAGTTCGTGCGGTTCGACAGCGACTCGGCGTGTCCGAGGATGGAGCCGCGGGCGCCGTGGGTGGAGCAGGAGGGGCCGGAGTATTGGGAAGAGGAGACACGGAACACCAAGGCCCACGCACAGACTGACAGAATGAACCTGCAGACC
CTGCGCGGCTACTACAACCAGAGCGAGGCCAGTTCTCACACCCTCCAGTGGATGATTGGCTGCGACCTGGGGTCCGACGGACGCCTCCTCCGCGGGTATGAACAGTATGCCTACGATGGCAAGGATTACCTCGCCCTGAACGAGGACCTGCGCTCCTG
GACCGCAGCGGACACTGCGGCTCAGATCTCCAAGCGCAAGTGTGAGGCGGCCAATGTGGCTGAACAAAGGAGAGCCTACCTGGAGGGCACGTGCGTGGAGTGGCTCCACAGATACCTGGAGAACGGGAAGGAGATGCTGCAGCGCGCGGACCCCC
CCAAGACACACGTGACCCACCACCCTGTCTTTGACTATGAGGCCACCCTGAGGTGCTGGGCCCTGGGCTTCTACCCTGCGGAGATCATACTGACCTGGCAGCGGGATGGGGAGGACCAGACCCAGGACGTGGAGCTCGTGGAGACCAGGCCTGCAGG
GGATGGAACCTTCCAGAAGTGGGCAGCTGTGGTGGTGCCTTCTGGAGAGGAGCAGAGATACACGTGCCATGTGCAGCATGAGGGGCTGCCGGAGCCCCTCATGCTGAGATGGAAGCAGTCTTCCCTGCCCACCATCCCCATCATGGGTATCGTTGCT
GGCCTGGTTGTCCTTGCAGCTGTAGTCACTGGAGCTGCGGTCGCTGCTGTGCTGTGGAGAAAGAAGAGCTCAGATTGA

>CDS_HLA-G_010401
ATGGTGGTCATGGCACCCCGAACCCTCTTCCTGCTACTCTCGGGGGCCCTGACCCTGACCGAGACCTGGGCGGGCTCCCACTCCATGAGGTATTTCAGCGCCGCCGTGTCCCGGCCCGGCCGCGGGGAGCCCCGCTTCATCGCCATGGGCTACGTGGA
CGACACGCAGTTCGTGCGGTTCGACAGCGACTCGGCGTGTCCGAGGATGGAGCCGCGGGCGCCGTGGGTGGAGCAGGAGGGGCCAGAGTATTGGGAAGAGGAGACACGGAACACCAAGGCCCACGCACAGACTGACAGAATGAACCTGCAGACC
CTGCGCGGCTACTACAACCAGAGCGAGGCCAGTTCTCACACCCTCCAGTGGATGATTGGCTGCGACCTGGGGTCCGACGGACGCCTCATCCGCGGGTATGAACAGTATGCCTACGATGGCAAGGATTACCTCGCCCTGAACGAGGACCTGCGCTCCTG
GACCGCAGCGGACACTGCGGCTCAGATCTCCAAGCGCAAGTGTGAGGCGGCCAATGTGGCTGAACAAAGGAGAGCCTACCTGGAGGGCACGTGCGTGGAGTGGCTCCACAGATACCTGGAGAACGGGAAGGAGATGCTGCAGCGCGCGGACCCCC
CCAAGACACACGTGACCCACCACCCTGTCTTTGACTATGAGGCCACCCTGAGGTGCTGGGCCCTGGGCTTCTACCCTGCGGAGATCATACTGACCTGGCAGCGGGATGGGGAGGACCAGACCCAGGACGTGGAGCTCGTGGAGACCAGGCCTGCAGG
GGATGGAACCTTCCAGAAGTGGGCAGCTGTGGTGGTGCCTTCTGGAGAGGAGCAGAGATACACGTGCCATGTGCAGCATGAGGGGCTGCCGGAGCCCCTCATGCTGAGATGGAAGCAGTCTTCCCTGCCCACCATCCCCATCATGGGTATCGTTGCT
GGCCTGGTTGTCCTTGCAGCTGTAGTCACTGGAGCTGCGGTCGCTGCTGTGCTGTGGAGGAAGAAGAGCTCAGATTGA

>CDS_HLA-G_0106
ATGGTGGTCATGGCACCCCGAACCCTCTTCCTGCTACTCTCGGGGGCCCTGACCCTGACCGAGACCTGGGCGGGCTCCCACTCCATGAGGTATTTCAGCGCCGCCGTGTCCCGGCCCGGCCGCGGGGAGCCCCGCTTCATCGCCATGGGCTACGTGGAC
GACACGCAGTTCGTGCGGTTCGACAGCGACTCGGCGTGTCCGAGGATGGAGCCGCGGGCGCCGTGGGTGGAGCAGGAGGGGCCAGAGTATTGGGAAGAGGAGACACGGAACACCAAGGCCCACGCACAGACTGACAGAATGAACCTGCAGACCC
TGCGCGGCTACTACAACCAGAGCGAGGCCAGTTCTCATACCCTCCAGTGGATGATTGGCTGCGACCTGGGGTCCGACGGACGCCTCCTCCGCGGGTATGAACAGTATGCCTACGATGGCAAGGATTACCTCGCCCTGAACGAGGACCTGCGCTCCTGG
ACCGCAGCGGACACTGCGGCTCAGATCTCCAAGCGCAAGTGTGAGGCGGCCAATGTGGCTGAACAAAGGAGAGCCTACCTGGAGGGCACGTGCGTGGAGTGGCTCCACAGATACCTGGAGAACGGGAAGGAGATGCTGCAGCGCGCGGACCCCCC
CAAGACACACGTGACCCACCACCCTGTCTTTGACTATGAGGCCACCCTGAGGTGCTGGGCCCTGGGCTTCTACCCTGCGGAGATCATACTGACCTGGCAGCGGGATGGGGAGGACCAGACCCAGGACGTGGAGCTCGTGGAGACCAGGCCTGCAGG
GGATGGAACCTTCCAGAAGTGGGCAGCTGTGGTGGTGCCTTCTGGAGAGGAGCAGAGATACATGTGCCATGTGCAGCATGAGGGGCTGCCGGAGCCCCTCATGCTGAGATGGAAGCAGTCTTCCCTGCCCACCATCCCCATCATGGGTATCGTTGCT
GGTCTGGTTGTCCTTGCAGCTGTAGTCACTGGAGCTGCGGTCGCTGCTGTGCTGTGGAGGAAGAAGAGCTCAGATTGA

Supplementary figure 3 : The nucleotide sequences of HLA-G*01:01, *01:04 and *01:06

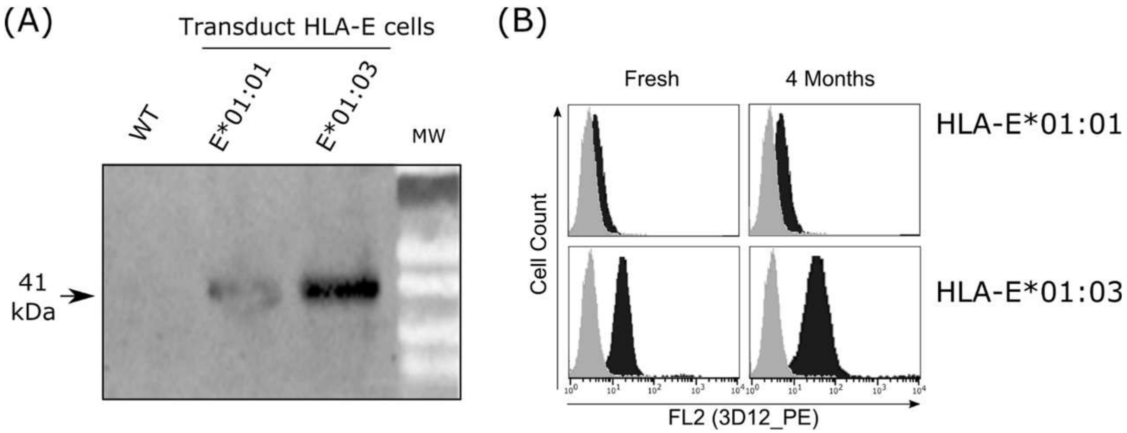

Supplementary figure 4: Expression of HLA-E isoform on SPI801 cell lines. (A) Western Blot analysis of HLA-E in the transduced cells in comparison with non-transduced cells (WT = wild type). (B) Stable expression levels of HLA-E isoform in transduced cells (black) versus non-transduced cells (gray) by flow cytometry.

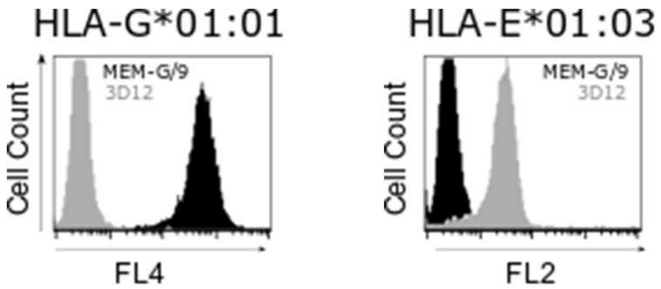

Supplementary figure 5: Specificity of detection of HLA-G and HLA-E by MEMG/9 and 3D12, respectively. Histograms show flow cytometry analysis of cells stained with MEM-G/9 (anti HLA-G antibody) in black or 3D12 (anti HLA-E antibody) in grey. Left histogram shows absence of detection of HLA-E on HLA-G*01:01 cells. Right histogram shows absence of detection of HLA-G on HLA-E*01:03 cells. Same analysis was performed on HLA-G*01:04, HLA-G*01:06 and HLA-E*01:01cells line (Data not shown).
