# Supplementary material for: Low Prevalence of HLA-G Antibodies in Lung Transplant Patients Detected using MAIPA-Adapted Protocol
Source: Int J Mol Sci. 2023 Nov 18;24(22):16479. doi: 10.3390/ijms242216479 (PMC10671704; doi:10.3390/ijms242216479)
Supplement: Supplementary file 1 [file ijms-24-16479-s001.zip › Table S1.pdf]

Table S1. Results of DSA in LTRs cohort at D0, D15, M1, M3 and M12 after LTx

	Characteristic of Pathology							Collection Dates (D : Day ; M: Month)															HLA-G Typing	
								Do			D15			M1			M3			M12				
**Patients**	**Disease**	**CMV Status 0: absence mismatch ; 1:D+ et R- without seroconversion; 2:D+ et R- with seroconversion; 3:D- et R+**	**Type of LTx**	**HLA mismatch**	**Chronic ejection**	**Acute rejection**	**DSA**	**DSA classe I one lambda MFI**	**DSA classe II one lambda MFI**	**Ab non DSA**	**DSA classe I one lambda MFI**	**DSA classe II one lambda MFI**	**Ab non DSA**	**DSA classe I one lambda MFI**	DSA classe II one lambda MFI	Ab non DSA	DSA classe I one lambda MFI	DSA classe II one lambda MFI	Ab non DSA	DSA classe I one lambda MFI	DSA classe II one lambda MFI	Ab non DSA	Genotypes HLA-G	Haplotypes UTR
1	Histicocytosis	0	bilateral	8	Yes		D15	0	0	No	0	DQ3:10500<br>DQ5:2600		0	DQ5:8000<br>DQA03:9000	No	0	DQ3:5000	No	0	0	No	G*01:01,*--	UTR2, UTR4
2	Cystic fibrosis	0	bilateral	7				0	0	No	0	0		0	0	No	0	0	No	0	0	No	G*01:01,*--	UTR1, UTR1
3	Fibrosis	0	bilateral	8			M1	0	0	No	0	0		0	DQ7:4000	No	0	0	No	0	0	No	G*01:01,*--	UTR1, UTR1
4	Fibrosis	3	bilateral	0		M12	D15	0	0	No	0	DR7:2500<br>DQ2:1500		A30:4500<br>B13:8000<br>Cw4 ou CW5:2000	DR7:5000<br>DQ2:10000<br>DR53:3000	No	0	DQ2:5000,<br>DR53:4000	No	0	0	No	G*01:01,*--	UTR1, UTR1
5	Emphysema	0	single	3				0	0	No	0	0		0	0	No	0	0	No	0	0	No	G*01:01,*--	UTR1, UTR4

6	Cystic fibrosis	3	bilateral	8				0	0	No	0	0	0	0	Yes	0	0	No	0	0	No	G*01:01,*--	UTR1, UTR2
7	Emphysema	2	bilateral	6				0	0	No	0	0	0	0	No	0	0	No	0	0	No	G*01:01,G*01:06	UTR2, UTR6
8	Cystic fibrosis	0	bilateral	6			M1	0	0	No	0	0	B44:3300 A1:2400 B8:1500	DQ2:6300	No	0	0	No	0	0	No	G*01:01,G*01:04	UTR1, UTR3
9	Emphysema	0	bilateral	6			D0	B7:11000	B7:11000	No	B7:13000	0	B7:7000	0	No	B7:3000	0	No	B7:3000	0	No	G*01:01,G*01:06	UTR2, UTR2
10	Cystic fibrosis	0	bilateral	6			M3	0	0	No	0	0	0	0	Yes	A30:1500	DR7:5000 DQ2:12000 DR53:3000	No	0	0	No	G*01:01,G*01:04	UTR3, UTR6
11	Emphysema	0	bilateral	0			D15	0	0	No	B44:5000	0	0	0	No	0	0	No	0	0	No	G*01:01,G*01:06	UTR2, UTR2
12	Cystic fibrosis	0	single	8		M1	D15	0	0	No	A25:4500	DQ5:10000 DQ6:13000	0	DQ5:5000 DQ6:5000	No	0	0	No	0	0	No	G*01:01,G*01:05N	UTR4, UTR3
13	Cystic fibrosis	0	single	7			M1	0	0	No	0	0	B45:2000	DQ6:6000	No	0	0	No	0	0	No	G*01:01,*--	UTR1, UTR2
14	Fibrosis	0	bilateral	8			M1	0	0	No	0	0	0	DQ9:4900	Yes	0	0	No	0	0	No	G*01:01,G*01:04	UTR3, UTR7
15	Cystic fibrosis	0	bilateral	7			M1	0	0	No	0	0	B44:5000	DQ2:14000	No	0	0	No	0	0	No	G*01:01,*--	UTR1, UTR2
16	Fibrosis	2	single	7				0	0	No	0	0	0	0	No	0	0	No	0	0	No	G*01:01,*--	UTR2, UTR6
17	Fibrosis	0	bilateral	4			D15	0	0	No	0	DQ2:9000	0	DQ2:8000	No	0	DR7:4000 DQ2:12000	No	0	0	No	G*01:01,G*01:03	UTR1, UTR2

18	Cystic fibrosis	0	bilateral	5			M1	0	0	No	0	0	A2:3100, B44:2900	0	Yes	0	0	No	0	0	No	G*01:01,*--	UTR4, UTR2
19	Cystic fibrosis	0	bilateral	8			D15	0	0	Yes	0	DQ5:9500 DQ6:9500	0	DQ5:7500 DQ6:6000	Yes	0	DQ5:3500	No	0	0	No	G*01:03,G*01:06	UTR2, UTR5
20	Cystic fibrosis	0	single	6				0	0	No	0	0	0	0	No	0	0	No	0	0	No	G*01:01,*--	UTR1, UTR6
21	Fibrosis	0	bilateral	0	Yes	M3	M1	0	0	No	0	0	0	DQ2:13000	No	0	DQ2:4500	No	0	DQ2:6000	No	G*01:01,G*01:04	UTR4, UTR3
22	Emphysema	2	bilateral	5	Yes		D15	0	0	Yes	0	DQ4:13000 DR8:4000	B58:2000	DQ4:15000	No	0	DQ4:5000	Yes	0	0	Yes	G*01:01,*--	UTR1, UTR2
23	Bronchial Dysplasia	3	bilateral	7	Yes		D0	0	0	No	0	DQ7:9000	0	DQ7:9000	No	0	DQ7:9000	Yes	0	0	No	G*01:01,*--	UTR4, UTR6
24	Cystic fibrosis	0	bilateral	0				0	0	No	0	0	0	0	No	0	0	No	0	0	No	G*01:01,G*01:05N	UTR1, UTR2
25	Fibrosis	0	single	6				0	0	No	0	0	0	0	No	0	0	No	0	0	No	G*01:01,*--	UTR4, UTR7
26	Cystic fibrosis	0	bilateral	6			D15	0	0	No	0	DQ7:12000 DR12:6000 DR52:2500 DR11:1500	0	DQ7:16000 DR12:3500 DR52:1700	No	0	0	Yes	0	0	No	G*01:01,*--	UTR1, UTR2
27	Emphysema	0	single	7	Yes	M12	M1	0	0	No	A2:9000	DR8:8000	A23:12000	0	No	0	0	No	0	0	No	G*01:01,*--	UTR1, UTR2
28	Cystic fibrosis	3	bilateral	7		M3	M1	0	0	No	0	0	A2:7000 A24:5	DQ2:12000	No	0	DQ2:10000	No	0	DQ2:6000	No	G*01:01,*--	UTR2, UTR4

														000 B44:3 000 B38:1 5000	DR53:2 550									
29	Cystic fibrosis	1	bilat eral	7				0	0	No	0	0		0	0	No	0	0	No	0	0	No	G*01:01,G* 01:04	UTR1, UTR3
30	Cystic fibrosis	2	bilat eral	7		M3	M 1	0	0	No	0	0		A26:1 2000 A3:85 00 B7:85 00	DQ4:18 500, DR8:41 00, DR15:4 000	No	0	DQ4:1 6000	Ye s	0	0	No	G*01:01,*--	UTR1, UTR2
31	Cystic fibrosis	0	bilat eral	8	Yes		M 1	0	0	No	0	0		B8:36 00	DQ2:11 000	No	B8: 2000	0	Ye s	0	0	Ye s	G*01:01,*--	UTR7, UTR1
32	Fibrosis	3	singl e	6			D0	B27:6 000	B27:6 000	No	0	0		0	0	No	0	0	No	0	0	No	G*01:01,G* 01:04	UTR1, UTR3
33	Cystic fibrosis	0	bilat eral	0		M3	M 1	0	0	No	0	0		A31:2 000	0	No	0	0	Ye s	0	0	No	G*01:01,G* 01:06	UTR2, UTR2
34	Emphys ema	0	singl e	5			M 1	0	0	No	0	0		B8:13 000	DR17:1 4500 DQ7:13 000 DR4:45 00	No	0	0	No	0	0	No	G*01:01,*--	UTR1, UTR7
35	Fibrosis	0	bilat eral	7	Yes		M 1	0	0	No	0	0		A32: 7200	DQ9:58 00, DR12:5 500, DR7:19 00	No	A32: 5900 B57: 2000	0	No	0	0	No	G*01:01,*--	UTR1, UTR1
